# Correction: The morphology and surface charge-dependent cellular uptake efficiency of upconversion nanostructures revealed by single-particle optical microscopy

**DOI:** 10.1039/d2sc90037h

**Published:** 2022-03-08

**Authors:** Di Zhang, Lin Wei, Meile Zhong, Lehui Xiao, Hung-Wing Li, Jianfang Wang

**Affiliations:** State Key Laboratory of Medicinal Chemical Biology, Tianjin Key Laboratory of Biosensing and Molecular Recognition, College of Chemistry, Nankai University Tianjin 300071 China lehuixiao@nankai.edu.cn; Key Laboratory of Chemical Biology & Traditional Chinese Medicine Research, Key Laboratory of Phytochemical R&D of Hunan Province, College of Chemistry and Chemical Engineering, Hunan Normal University Changsha 410082 China; Department of Chemistry, Hong Kong Baptist University Kowloon Tong Hong Kong SAR China; Department of Physics, The Chinese University of Hong Kong Shatin Hong Kong SAR China

## Abstract

Correction for ‘The morphology and surface charge-dependent cellular uptake efficiency of upconversion nanostructures revealed by single-particle optical microscopy’ by Di Zhang *et al.*, *Chem. Sci.*, 2018, **9**, 5260–5269, DOI: 10.1039/C8SC01828F.

In the Experimental section of the article, the concentration of citrate was incorrectly given as 0.05 M. The correct concentration of citrate is 2 M.

The Royal Society of Chemistry apologises for these errors and any consequent inconvenience to authors and readers.

## Supplementary Material

